# Mutagenesis and Analysis for Enhancing (2*R*,3*R*)-2,3-Butanediol Tolerance and Production in *Corynebacterium glutamicum*

**DOI:** 10.3390/molecules31152714

**Published:** 2026-08-04

**Authors:** Zilong Liu, Haoran Cui, Yuxuan Jiang, Zhiwen Wang, Tao Chen

**Affiliations:** 1 State Key Laboratory of Synthetic Biology, Tianjin University, Tianjin 300072, China; lzl16630736892@163.com (Z.L.); cuihaoran@tju.edu.cn (H.C.); yuxuan_jiang@tju.edu.cn (Y.J.); 2 Frontier Science Center for Synthetic Biology and Key Laboratory of Systems Bioengineering (Ministry of Education), Tianjin University, Tianjin 300072, China; 3 School of Synthetic Biology and Biomanufacturing, Tianjin University, Tianjin 300072, China; 4 Key Laboratory of Ministry of Education for Protection and Utilization of Special Biological Resources in Western China, School of Life Sciences, Ningxia University, Yinchuan 750021, China

**Keywords:** 2,3-butanediol, *Corynebacterium glutamicum*, tolerance, ARTP mutagenesis, microbial fermentation, reverse engineering, cytotoxicity

## Abstract

2,3-Butanediol (2,3-BD) is a versatile bulk chemical, serving as a high-boiling green solvent, a key monomer for polyesters and polyurethanes, and a crucial intermediate in the production of fine chemicals and cosmetics. Additionally, it is a key intermediate for pharmaceuticals and agrochemicals, supporting green chemistry. Microbial production of 2,3-BD offers a sustainable bioprocess but faces the challenge of high-concentration product inhibition. In an effort to improve 2,3-BD tolerance and production in *Corynebacterium glutamicum*, the engineered strain CGK4 was subjected to atmospheric and room temperature plasma (ARTP) mutagenesis, from which three superior mutant strains were isolated. Genomic and reverse engineering analyses revealed two mutations beneficial for tolerance and seven mutations beneficial for production. Among these, mutations *atpG^L2^^50P^*, *cobT^V192V^*, and *ctaE^L79P^* increased 2,3-BD titer by 18.0%, 14.5%, and 15.0%, respectively. Furthermore, we found that while certain mutations markedly enhanced stress tolerance, the highest-producing strains achieved a balanced metabolic state rather than maximal tolerance, indicating a non-linear relationship between the two traits. Finally, through mutation combination optimization, the optimal strain K4-*cobT*-*ctaE* was constructed. In shake-flask fed-batch fermentation, this strain produced 84.35 ± 3.04 g/L (2*R*,3*R*)-2,3-BD, which was 38.3% higher than that of the parental strain. Our findings provide a promising approach to addressing 2,3-BD tolerance challenges and promote the development of microbial production platforms for industrial application.

## 1. Introduction

2,3-Butanediol (2,3-BD) is an industrially valuable organic compound that exists as three stereoisomers: (2*R*,3*R*)-, (2*S*,3*S*)-, and *meso*-2,3-BD [1,2]. As a bulk chemical, it serves as a key synthetic intermediate for various chemicals [3] and is widely used as a fuel additive, antifreeze agent, and plant growth regulator [4,5], offering broad application prospects and significant market potential.

Traditional 2,3-BD production relies on energy-intensive, high-cost chemical synthesis from petroleum, which also causes environmental pollution. Therefore, sustainable microbial production of 2,3-BD has gained increasing attention in recent years [6]. Progress in 2,3-BD biosynthesis has been achieved in *Klebsiella* spp. [7,8], *Bacillus* spp. [9,10], *Enterobacter* spp. [11,12], *Saccharomyces cerevisiae* [13,14], and *Escherichia coli* [15,16].

As a generally recognized as safe (GRAS) microorganism, *Corynebacterium glutamicum* exhibits superior biosafety, enhanced robustness, and broader carbon source utilization compared to other chassis strains [17]. A previous study reported the construction of the engineered strain CGK4 via heterologous pathway integration and systematic metabolic engineering, which enabled the biosynthesis of (2*R*,3*R*)-2,3-BD in *C. glutamicum* [18]. However, consistent with most current studies, high-level 2,3-BD fermentation is limited by product inhibition, restricting further improvements in titer (defined as product concentration in g/L). Therefore, improving strain tolerance to high concentrations of 2,3-BD has become a critical bottleneck requiring urgent breakthrough.

To date, studies on 2,3-BD tolerance remain scarce, with no reports available on tolerance in *C. glutamicum*. Exposure to 60 g/L (2*R*,3*R*)-2,3-BD resulted in near-complete arrest of cell proliferation and metabolic functions in *Paenibacillus polymyxa* [19]. Additionally, the toxicity of 2,3-BD varied among its stereoisomers, with the (2*R*,3*R*)-form exhibiting higher toxicity than the *meso*-form at equivalent concentrations. Researchers have also found that upon exceeding the tolerance threshold, 2,3-BD is converted back to its precursor, acetoin [19]. To enhance strain tolerance, random mutagenesis has been widely applied. Introducing DNA point and structural mutations significantly enhanced 2,3-BD tolerance in *S. cerevisiae* [20,21]. Transcriptomic analysis revealed that upregulation of genes involved in amino acid metabolism, proteasome, peroxisome, TCA cycle, mitochondria, and transcriptional regulation was associated with enhanced 2,3-BD tolerance. However, these studies only improved strain tolerance without directly achieving higher 2,3-BD production. Additionally, adaptive laboratory evolution (ALE) is another effective approach to enhance 2,3-BD tolerance. Through serial passaging under increasing 2,3-BD concentrations, tolerance in *Escherichia coli* was effectively enhanced [22]. Subsequent genomics and reverse engineering revealed multiple genes and key genetic regions highly associated with 2,3-BD tolerance. A proposed mechanism suggests that cells respond to 2,3-BD stress by modulating the cell membrane and transport across it, transcription and translation, and stress-related functional proteins. Nevertheless, only 10% of strains showed enhanced 2,3-BD titer, and evaluations were conducted only at low production levels without fed-batch fermentation for high-yield assessment.

Atmospheric and room temperature plasma (ARTP) is an efficient and safe novel mutagenesis technology that enables rapid construction of mutant libraries with greater genetic diversity [23]. ARTP mutagenesis combined with tolerance screening is an effective strategy for enhancing tolerance and breeding high-producing strains [24]. ARTP mutagenesis and salt screening produced *S. cerevisiae* HF-130 with 98.8% higher ethanol titer [25]. Iterative ARTP mutagenesis of *Bacillus pumilus* resulted in improved tolerance to gentamicin, lincomycin, and rifamycin, as well as a more than 80% enhancement in γ-polyglutamic acid yield [26]. Additionally, ARTP mutagenesis can be effectively combined with other breeding strategies. By combining ARTP mutagenesis with ALE, an *E. coli* with enhanced L-cysteine tolerance and titer was successfully developed [27].

**Figure 1:**
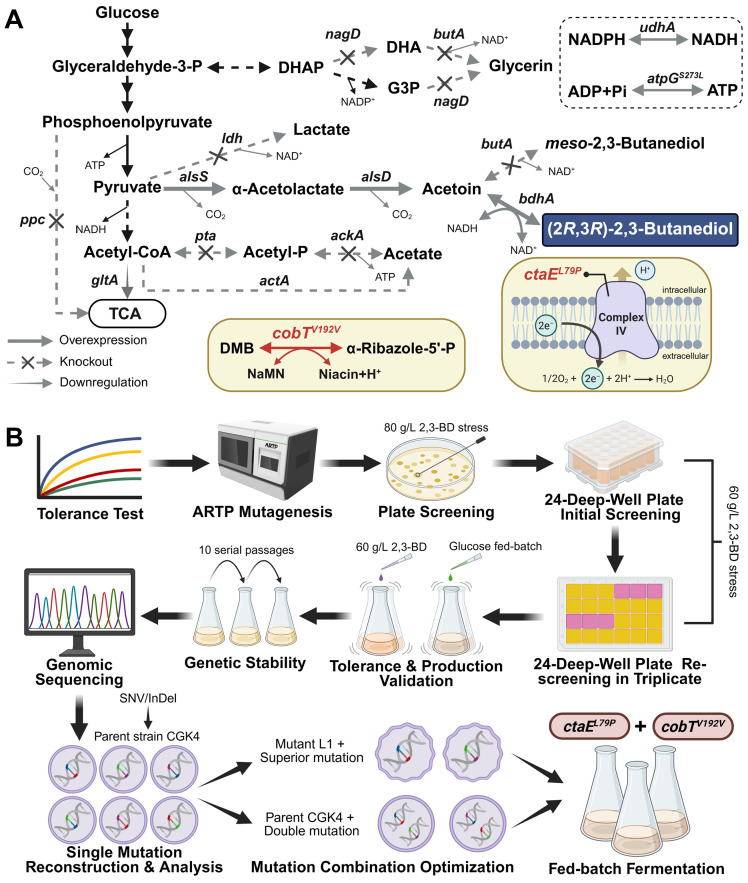
Scheme for engineering (2*R*,3*R*)-2,3-butanediol biosynthesis in *C. glutamicum.* (**A**) Metabolic network of strain K4-*cobT-ctaE* for (2*R*,3*R*)-2,3-butanediol production Gray arrows represent metabolic modifications in the background strain CGK4. Red indicates genomic mutations introduced in this study. Solid arrows represent the 2,3-butanediol biosynthesis pathway. Dashed arrows represent by-product biosynthesis pathways. Abbreviations: DHAP, dihydroxyacetone phosphate; DHA, dihydroxyacetone; G3P, *sn*-glycerol 3-phosphate; DMB, dimethylbenzimidazole; NaMN, nicotinate ribonucleotide; Complex IV, cytochrome c oxidase. Genes and their encoded enzymes: *alsS*, acetolactate synthase; *alsD*, acetolactate decarboxylase; *butA*, *meso*-2,3-butanediol dehydrogenase; *bdhA*, (2*R*,3*R*)-2,3-butanediol dehydrogenase; *nagD*, putative phosphatase; *ldh*, L-lactate dehydrogenase; *ppc*, phosphoenolpyruvate carboxylase; *gltA*, citrate synthase; *pta*, phosphotransacetylase; *ackA*, acetate kinase; *actA*, acetate CoA-transferase; *udhA*, transhydrogenase; *atpG*, H^+^-ATPase synthase subunit γ; *ctaE*, cytochrome c oxidase subunit III; *cobT*, nicotinate-nucleotide--dimethylbenzimidazole phosphoribosyltransferase. (**B**) Schematic of ARTP mutagenesis and reverse metabolic engineering for enhancing (2*R*,3*R*)-2,3-butanediol tolerance and production.

In this study, we aimed to enhance 2,3-butanediol production in an engineered strain by improving its tolerance to the product (Figure 1). ARTP mutagenesis was applied to the 2,3-BD producer *C. glutamicum* CGK4 [18], followed by screening for high-producing mutants under 2,3-BD tolerance pressure. Subsequent reverse engineering and mutation analysis identified multiple beneficial mutations associated with enhanced 2,3-BD tolerance or production. By optimizing the combination of elite mutations, we constructed the final strain K4-*cobT*-*ctaE*, which achieved a 38.3% higher 2,3-BD titer in shake-flask fed-batch fermentation relative to the parental strain. The stoichiometric theoretical maximum yield of 2,3-BD from glucose is 0.5 g/g (1 mol glucose → 1 mol 2,3-BD + 2CO_2_), which serves as the benchmark to evaluate the carbon conversion efficiency.

## 2. Results

### 2.1. High-Concentration 2,3-Butanediol Stress on C. glutamicum

To evaluate 2,3-BD stress on *C. glutamicum* and establish screening conditions for ARTP mutagenesis, the parental strain CGK4 was assessed for 2,3-BD tolerance on plates and in shake flasks. Growth on plates with different 2,3-BD concentrations is shown in Supplementary Figure S1. Increasing 2,3-BD concentrations resulted in gradual inhibition of cell growth, manifested by reduced colony numbers and slower growth rates. At 80 g/L 2,3-BD, colonies appeared only after 7 days, and the number was extremely low. At 100 g/L, no growth was observed. These results indicate that 80 g/L approximates the tolerance limit of the parental strain and represents the minimum inhibitory concentration. Consequently, this concentration was selected as the stringent screening pressure for plates after ARTP mutagenesis to isolate rare mutants with enhanced tolerance.

**Figure 2:**
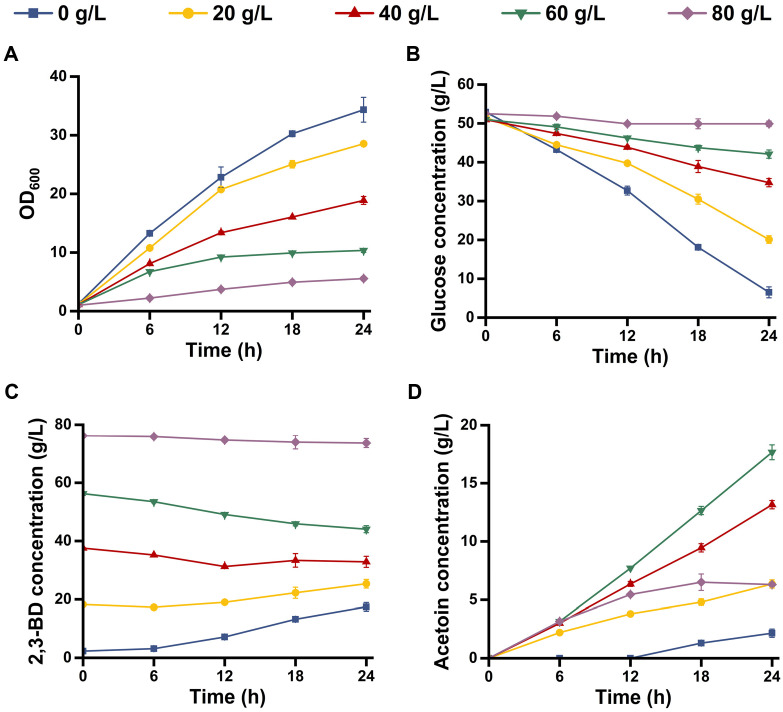
Effect of increasing 2,3-butanediol concentrations on CGK4 in shake flasks: (**A**) Growth. (**B**) Glucose consumption. (**C**) 2,3-Butanediol production. (**D**) Acetoin accumulation. Shake-flask fermentations were performed in CGXIIP medium with initial 0, 20, 40, 60, 80, or 100 g/L of 2,3-butanediol. Values and error bars represent the mean and SD (*n* = 3), respectively.

In shake-flask cultures with increasing 2,3-BD concentrations, CGK4 exhibited progressive inhibition of both growth and glucose consumption (Figure 2A,B). At 60 g/L 2,3-BD, metabolic activity was markedly inhibited, and 2,3-BD accumulation gradually decreased (Figure 2C). At 40 g/L, the strain began to consume 2,3-BD, presumably as a protective mechanism to alleviate toxicity by converting it to acetoin. This was supported by the progressively increasing acetoin accumulation (Figure 2D). At 80 g/L, strain growth was severely inhibited, and both glucose utilization and metabolite conversion were effectively halted. At 60 g/L, 2,3-BD inhibited growth and production but did not completely block physiological activity, indicating that this sub-lethal concentration preserves a selection window for distinguishing superior mutants. Consequently, 60 g/L was selected as a suitable concentration for mutant tolerance screening in liquid culture.

### 2.2. ARTP Mutagenesis of Strain CGK4 and Screening

Strengthening product tolerance to boost the production performance of cell factories constitutes a practical and effective strategy [26]. A lethality rate of approximately 90% in ARTP mutagenesis has been associated with maximal mutation diversity and optimal acquisition of positive mutations [28,29]. CGK4 was treated by ARTP mutagenesis with exposure times of 0, 30, 60, 90, 120, and 150 s, and the corresponding lethality rates were determined. When the exposure time reached 120 s, the lethality rate was 92.4% (Supplementary Figure S2). Accordingly, CGK4 was treated by ARTP mutagenesis under the optimal conditions of 120 W, 10 SLM, and 120 s to obtain a mutant library enriched for improved 2,3-BD tolerance and production.

After ARTP mutagenesis, the resulting strains showed considerable differences in growth and production under high 2,3-BD stress, facilitating the screening of high-performance mutants. From the ARTP-mutagenized library, 240 single colonies were preliminary selected using plates containing 80 g/L 2,3-BD. Subsequently, the single colonies were inoculated into 24-deep-well plates containing 60 g/L 2,3-BD, and OD_600_ and 2,3-BD titers were measured at the end of fermentation (Supplementary Figure S3A). The top 60 mutants with the highest 2,3-BD accumulation were selected for secondary screening in deep-well plates, with three parallel replicates per strain (Supplementary Figure S3B). The ten mutants with the highest 2,3-BD titer were selected as candidates for final testing.

### 2.3. Fermentation Evaluation of Mutant Strains

**Figure 3:**
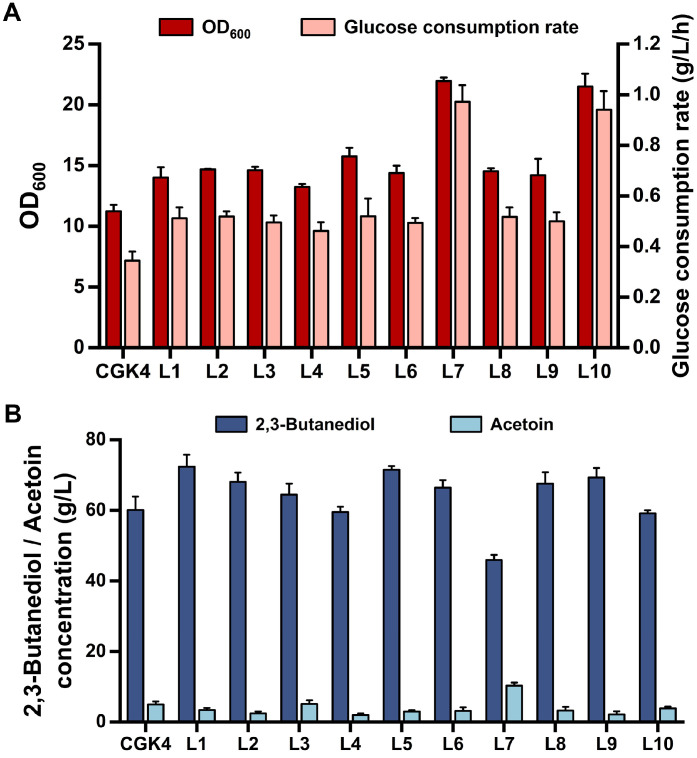
Evaluation of strains L1 to L10 and CGK4: (**A**) OD_600_ and glucose consumption rate of shake-flask cultures at 24 h under 60 g/L 2,3-butanediol stress. (**B**) 2,3-Butanediol and acetoin titers at 120 h during shake-flask fed-batch fermentation. Values and error bars represent the mean and SD (*n* = 3), respectively.

The 10 mutants were designated L1 to L10 and systematically evaluated in shake flasks for tolerance, production and stability. OD_600_ under stress increased by over 15% in all ten mutant strains, with the glucose consumption rate following a similar pattern (Figure 3A). These results indicate that ARTP mutagenesis successfully improved strain tolerance and metabolic activity under high 2,3-BD conditions. Notably, strains L7 and L10 had 95.6% and 91.5% higher OD_600_ under stress than CGK4, respectively, while glucose consumption rate increased by over 170% in both strains.

Moreover, fed-batch fermentation with longer duration allows higher 2,3-BD titers, making it more suitable for evaluating the overall performance of strains in sustained fermentation (Supplementary Figure S4). Six mutant strains showed an increase in 2,3-BD production of more than 10% compared with CGK4 (Figure 3B). Among them, L1, L5, and L9 produced 72.96 ± 3.38, 72.10 ± 1.04, and 69.86 ± 2.72 g/L of 2,3-BD, representing increases of 21.4%, 20.0%, and 16.2%, respectively. Collectively, the results indicate that the tolerance of L1, L5, and L9 was enhanced by 25% to 40%, thereby validating that improving product tolerance effectively facilitates product synthesis. In contrast, although L7 and L10 displayed the most significant enhancement in tolerance, their production levels declined. Studies have shown that excessive enhancement of yeast tolerance may lead to reduced fermentation performance [30]. We therefore speculate that within a certain range, 2,3-BD production correlates positively with tolerance, but beyond a threshold, excessive tolerance impairs production [31]. Moderate enhancement of tolerance may be more favorable for 2,3-BD synthesis.

The genetic and production stability of L1, L5, and L9 was verified through serial passaging and repeated fermentation. After 10 generations, 2,3-BD production remained similar to that of the original strains (Supplementary Figure S4D), and no reversion mutations were detected, indicating good production stability under serial passage conditions.

### 2.4. Validation and Analysis of Mutations by Reverse Engineering

**Figure 4:**
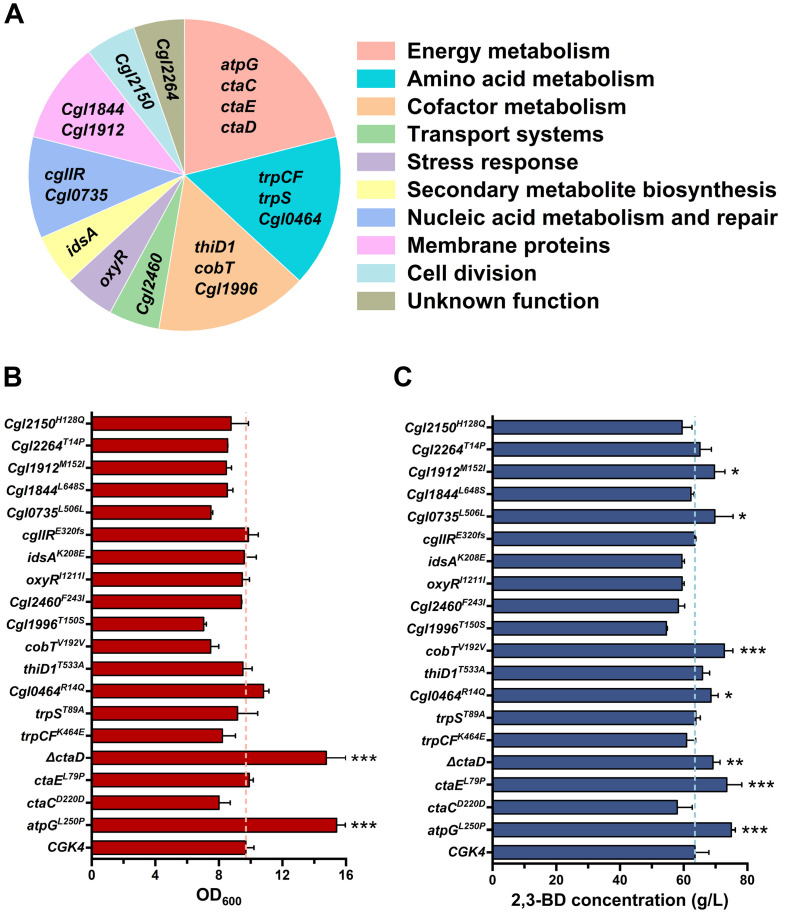
Validation and analysis of single mutation effects: (**A**) Functional classification of all mutated genes. (**B**) OD_600_ of shake-flask cultures at 24 h under 60 g/L 2,3-butanediol stress. (**C**) 2,3-Butanediol titer at 120 h during shake-flask fed-batch fermentation. Dashed lines represent the OD_600_ or titer values of the control strain CGK4. Values and error bars represent the mean and SD (*n* = 3), respectively. * *p* < 0.05, ** *p* < 0.01, *** *p* < 0.001.

Using genomics to obtain genetic variation information of mutant strains is essential for expanding genomic knowledge of strains and applying it to different strains. The three top-producing mutants, L1, L5, and L9, were subjected to genome resequencing. Comparative genomic analysis with the parental strain CGK4 revealed a total of 19 SNV/InDel mutations across the three mutant genomes (Table 1). ARTP mutagenesis tends to generate diverse SNVs and small InDels, thereby effectively improving strain characteristics [32]. Functional annotation and classification of the mutations according to cellular functions revealed that they were mainly enriched in genes involved in energy metabolism, amino acid metabolism, cofactor metabolism, membrane proteins, and nucleic acid metabolism and repair (Figure 4A). These results suggest that mutations in these gene categories may play key roles in enhancing 2,3-BD production or tolerance.

Considering that not all mutations are effective, reverse metabolic engineering was used to reconstruct each mutation individually in CGK4, generating 19 single-mutation strains to explore the relationship between genotype and phenotype. The frameshift mutation at the *ctaD* locus severely hindered normal protein expression; therefore, the *ctaD* gene was directly knocked out to verify the effect of the mutation instead. The effect of each single mutation on tolerance was tested in shake-flask fermentation initially containing 60 g/L 2,3-BD. The results showed that mutations *atpG^L250P^* and Δ*ctaD* significantly improved 2,3-BD tolerance in the single-mutant strains, increasing OD_600_ under stress by 58.9% and 52.2% compared with CGK4, respectively (Figure 4B). Other mutations had minor or adverse effects on tolerance.

Subsequently, all single-mutant strains were subjected to fed-batch fermentation in shake flasks to evaluate the effect of each single mutation on 2,3-BD synthesis. Seven beneficial mutations that significantly increased 2,3-BD production were identified, including *atpG^L250P^*, *cobT^V192V^*, *Cgl0464^R14Q^*, *ctaE^L79P^*, *Cgl0735^L506L^*, *Cgl1912^M152I^* and Δ*ctaD* (Figure 4C). Among them, *atpG^L250P^*, *cobT^V192V^*, and *ctaE^L79P^* showed the most pronounced effects. The single-mutant strains carrying these mutations produced 74.95, 72.73, and 73.53 g/L of 2,3-BD, respectively, corresponding to increases of 18.0%, 14.5%, and 15.0% relative to CGK4. Other mutations had little or negative effects on production.

### 2.5. Mutation Combination Optimization for Efficient Production of (2R,3R)-2,3-Butanediol

Reverse metabolic engineering via single-mutation reconstruction revealed the effects of each mutant genotype on tolerance and production phenotypes. However, some mutation combinations may exert synergistic effects, while combining other mutations may lead to detrimental outcomes. Moreover, a single mutation has limited capacity to improve strain production performance. To investigate whether the combined effect of beneficial mutations could lead to even higher production, we optimized mutation combinations using two strategies: combining mutant strains with additional mutation sites, and constructing double mutations of high-efficiency sites.

**Figure 5:**
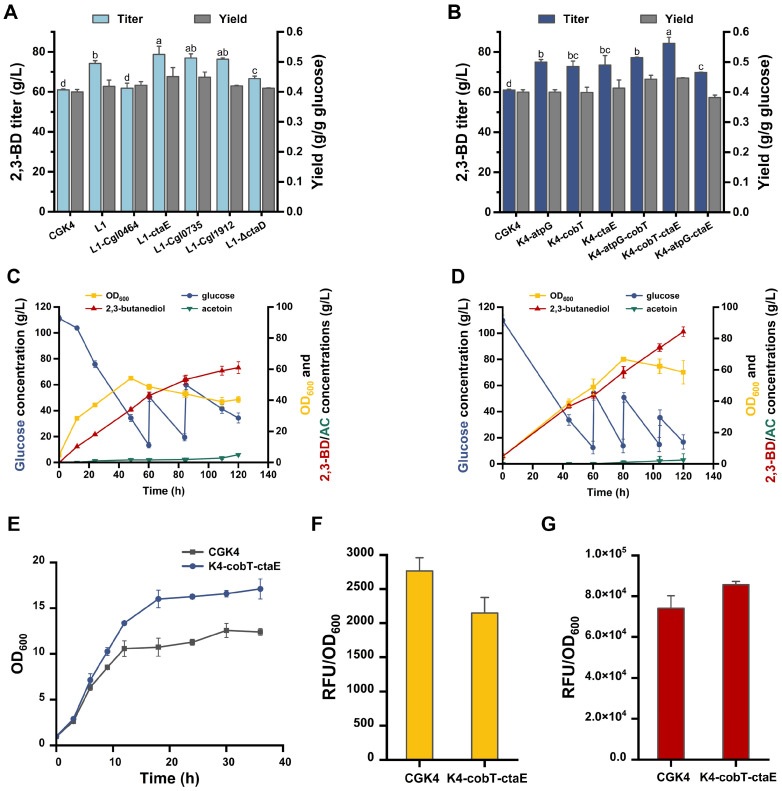
Mutation combination for efficient 2,3-butanediol production: (**A**) Effect of mutant L1 and mutation combinations on 2,3-butanediol production. (**B**) Effect of dual elite mutation combinations on 2,3-butanediol production. Metabolic profiles of strain (**C**) CGK4 and (**D**) K4-*cobT-ctaE* in shake-flask fed-batch fermentation. Comparison of (**E**) strain growth, (**F**) PI staining, and (**G**) FDA staining between CGK4 and K4-*cobT-ctaE* under 60 g/L 2,3-BD stress. Fluorescence intensity was expressed as relative fluorescence units (RFU) normalized to OD_600_. Values and error bars represent the mean and SD (*n* = 3), respectively.

In the mutant strain + mutation strategy, beneficial mutations from other mutants were introduced into an elite strain. L1, which already carried two beneficial mutations (*atpG^L250P^* and *cobT^V192V^*), showed the best improvement in 2,3-BD production. Using L1 as the chassis, we individually introduced the effective mutations from L5 and L9, including *Cgl0464^R14Q^*, *ctaE^L79P^*, *Cgl0735^L506L^*, *Cgl1912^M152I^* and Δ*ctaD*, to construct five combined strains, which were then evaluated in shake-flask fed-batch fermentation. Strain L1-*ctaE* achieved the highest 2,3-BD titer of 78.73 ± 4.06 g/L, which was 6.0% higher than L1 and 29.0% higher than CGK4 (Figure 5A). This indicates a synergistic effect between *ctaE^L79P^* and these mutations in L1. The mutant strain + mutation strategy successfully enhanced 2,3-BD production. Notably, *Cgl0735^L506L^* and *Cgl1912^M152I^* did not bring obvious gains, possibly because their effects overlapped with those of mutations already present in L1. The introduction of *Cgl0464^R14Q^* and Δ*ctaD* instead reduced titer by 16.8% and 10.3%, respectively, compared with L1. We speculate that a simple combination of beneficial factors does not always produce a positive effect, which may involve complex factors such as increased metabolic burden, imbalanced energy allocation, or disruption of regulatory networks.

Another strategy was a double-mutation combination. The mutations *atpG^L250P^*, *cobT^V192V^* and *ctaE^L79P^* were identified as the most beneficial mutations for improving 2,3-BD production. A single mutation already increased titer by more than 10%. Could a combination of two mutations produce a synergistic effect? Moreover, combining high-efficiency mutations may also help avoid interference from deleterious mutations. Accordingly, we introduced each of the three pairwise mutation combinations into CGK4 and performed shake-flask fed-batch fermentation. Strain K4-*cobT-ctaE* significantly increased 2,3-BD production from 61.01 ± 0.42 g/L to 84.35 ± 3.04 g/L, representing a 38.3% increase over CGK4. Strain K4-*cobT-ctaE* consumed 188.7 g/L glucose within 120 h, with a 2,3-BD yield of 0.447 g/g (89.4% of the theoretical yield), whereas CGK4 only gave a yield of 0.400 g/g glucose (Figure 5B). Notably, the production increase exceeded the sum of the increases from the two corresponding single-mutation strains, achieving a “1 + 1 > 2” effect. Although K4-*atpG-cobT* and K4-*atpG-ctaE* increased titer by 26.6% and 14.3%, respectively, neither showed a significant difference from their corresponding single-mutant strains, indicating that the combinations did not confer further improvement. We speculate that both mutations enhance the strain through energy synthesis, and their combination may involve overlapping regulatory pathways, making synergy difficult to achieve.

We further evaluated the tolerance of K4-*cobT-ctaE*. Figure 5E shows the growth under 60 g/L 2,3-BD compared with CGK4. During the logarithmic phase, K4-*cobT-ctaE* exhibited a clear growth advantage with accelerated cell proliferation and also reached a higher OD_600_ in the stationary phase, indicating enhanced tolerance. In addition, we stained stressed cells with propidium iodide (PI) and fluorescein diacetate (FDA). PI serves as a marker for membrane integrity and cell death; damaged or dead cells show higher fluorescence. K4-*cobT-ctaE* showed a 31.3% lower RFU/OD_600_ than CGK4 (Figure 5F), suggesting better membrane integrity against 2,3-BD toxicity. FDA labels metabolically active cells to reflect viability. K4-*cobT-ctaE* had a 15.7% higher RFU/OD_600_ than CGK4 (Figure 5G), indicating greater cell viability under stress. In fed-batch fermentation, when 2,3-BD accumulated above 40 g/L, K4-*cobT-ctaE* also showed more sustained growth, a higher glucose consumption rate and total utilization (Figure 5C,D). This indicates that K4*-cobT-ctaE* maintains superior metabolic activity during the gradual build-up of product toxicity, and its enhanced tolerance promotes more efficient carbon uptake for energy and precursor supply for growth and production. Collectively, *cobT^V192V^* and *ctaE^L79P^* moderately improved 2,3-BD tolerance. Notably, each mutation alone did not improve tolerance, but their combination produced an unexpected synergistic effect, indicating strong cooperativity in tolerance enhancement.

Through two different approaches of mutation combination optimization, we identified K4-*cobT-ctaE* as the best engineered strain in this study. This strain overcame high product toxicity inhibition in fed-batch fermentation and further improved 2,3-BD production. The results reveal that the *cobT^V192V^* and *ctaE^L79P^* combination has an additive effect, and this combination may act synergistically in energy metabolism and cofactor utilization.

## 3. Discussion

In industrial fermentation, high-level 2,3-BD production is challenged by product toxicity. Due to limitations in gene editing tools and insufficient knowledge of microbial genomes, traditional rational metabolic engineering approaches are often ineffective and fail to comprehensively improve cell factory performance. In this study, by combining ARTP mutagenesis, genomic analysis, and reverse engineering, we enhanced product tolerance in *Corynebacterium glutamicum* and constructed a high-level 2,3-BD strain, K4-*cobT-ctaE*. We also identified several efficient stress-resistant and production-related mutation targets. Strain K4-*cobT-ctaE* produced 84.35 ± 3.04 g/L (2*R*,3*R*)-2,3-BD in 120 h of shake-flask fed-batch fermentation, achieving 89.4% of the theoretical yield (Table 2).

By evaluating the tolerance of the parental strain CGK4, we confirmed that high concentrations of 2,3-BD significantly inhibit cell growth and metabolic activity. As 2,3-BD concentration increased, both growth and glucose consumption rate gradually slowed. Moreover, when the concentration exceeded a certain threshold (40 g/L), the strain began to consume 2,3-BD and convert it to acetoin (Figure 2C,D). We speculate that this is a protective mechanism employed by the strain to alleviate toxicity. Okonkwo reported that the reversal of 2,3-BD to acetoin is an active detoxification response in *Paenibacillus polymyxa* [19]. This behavior may help reduce 2,3-BD stress, or the stress may alter the intracellular NAD^+^/NADH balance, shifting the reaction toward decomposition. Yeast sacrifices growth and fermentation capacity to gain higher ethanol tolerance [41]. After acquiring lactate tolerance, *Zymomonas mobilis* halved its lactate production due to downregulation of key genes [42]. Furthermore, during tolerance screening of mutagenized strains, we observed that some strains exhibited high OD_600_ values but showed negative 2,3-BD accumulation (Supplementary Figure S3A). This further confirms the phenomenon of alleviating toxic stress at the expense of 2,3-BD synthesis. We postulate that this observation is governed by a diffusion-thermodynamic equilibrium: the total extracellular concentration of (2*R*,3*R*)-2,3-BD and acetoin determines the transmembrane gradient, while the reversible interconversion (acetoin ↔ 2,3-BD) acts as a buffering mechanism to alleviate oxidative and osmotic stress. Once the total pool concentration surpasses a critical threshold, the thermodynamic driving force for net 2,3-BD synthesis diminishes, resulting in a pseudostationary state of metabolite distribution.

The balance between improved product tolerance and production also drew our attention. Supplementary Figure S3A shows that strains with low 2,3-BD accumulation mostly grew poorly. In contrast, top accumulators exhibited moderate rather than maximal growth, suggesting that the positive correlation between tolerance and production may hold only within a certain range. Similarly, excessively high tolerance in the re-screened strains did not lead to maximal production improvement (Supplementary Figure S3B), further supporting our finding. We speculate that under high 2,3-BD stress, the strain must balance product toxicity relief with metabolic activity. By allocating excessive resources to enhance tolerance, the strain diverts less material and energy to the product synthesis pathway, leading to reduced production performance [19]. Our subsequent mutation combination engineering further indicated that a direct causality between enhanced tolerance and elevated titer was not universally observed. Although Δ*ctaD* and *atpG^L250P^* conferred the most significant improvements in stress biomass (Figure 4B), their introduction into the L1 chassis (L1-Δ*ctaD*) or combination with other mutations (K4-*atpG-ctaE*) failed to yield proportional increases in 2,3-BD production (Figure 5A,B). This suggests that excessive reinforcement of stress-defense mechanisms imposes a metabolic burden that diverts carbon and energy fluxes away from product biosynthesis.

Recently, using ARTP mutagenesis to enhance strain tolerance to various stresses has become an important research area in microbial breeding [21]. Our study confirmed that ARTP mutagenesis combined with tolerance screening yields *C. glutamicum* strains with both high 2,3-BD tolerance and production, and these superior traits are maintained after multiple passages, demonstrating the great potential of ARTP technology for enhancing strain tolerance. Mutation analysis in this study identified two mutations (*atpG^L250P^* and Δ*ctaD*) that enhance 2,3-BD tolerance, and seven mutations (*atpG^L250P^*, *cobT^V192V^*, *Cgl0464^R14Q^*, *ctaE^L79P^*, *Cgl0735^L506L^*, *Cgl1912^M152I^* and Δ*ctaD*) that boost 2,3-BD production. Genomic analysis revealed that the mutations were mainly concentrated in functional genes involved in energy metabolism, amino acid metabolism, cofactor metabolism, membrane proteins, and nucleic acid metabolism and repair (Figure 4A). This is similar to the genetic regions associated with 2,3-BD stress response models reported in other strains [20]. Our study also provides a reference for understanding the mechanisms of 2,3-BD tolerance.

Among the effective mutations, the three most beneficial ones (*atpG^L250P^*, *cobT^V192V^* and *ctaE^L79P^*) drew our special attention. The *atp* operon encodes the F_0_F_1_-ATPase. The γ subunit of the membrane-embedded proton channel F_1_ is encoded by *atpG*. A defect in the γ subunit leads to proton leakage [43]. Sekine reported that an ATPase mutant with 30% reduced activity still showed increased glucose consumption [44]. We speculate that the *atpG* mutation may reduce cellular energy synthesis efficiency, thereby accelerating glycolysis to secure energy supply, while also increasing the accumulation of pyruvate and its downstream metabolites. Mutations in *atpG* have often been found to be closely associated with tolerance [45]. The *cobT* gene encodes phosphoribosyltransferase, a key enzyme in the cobalamin (vitamin B_12_) biosynthesis pathway. As a coenzyme indirectly involved in the TCA cycle, cobalamin plays important roles in carbon flux regulation, redox reactions, and stress responses [46]. Lee demonstrated that disruption of cobalamin synthesis leads to reduced reactive oxygen species (ROS) production and enhanced antibiotic tolerance [47]. The synonymous mutation in *cobT* may downregulate its expression via codon bias or mRNA secondary structure, thereby weakening the TCA cycle and ultimately increasing the pyruvate pool. In addition, the *cobT* mutation also affects the NAD^+^/NADH balance [48], which may in turn influence 2,3-BD synthesis. The *ctaE* gene encodes subunit III of cytochrome *aa_3_* oxidase, which transfers electrons from cytochrome c to oxygen, generating water, and establishes a proton gradient via proton pumping to drive adenosine triphosphate (ATP) synthesis [49]. In oxygen-limited environments, cytochrome *bd* oxidase replaces cytochrome *aa_3_* oxidase, and the cell shifts to a less efficient respiratory chain [50]. The *ctaE* mutation may reduce cytochrome *aa_3_* oxidase activity, forcing the cell to rely on the less efficient cytochrome *bd* oxidase-mediated respiratory chain. This leads to lower ATP synthesis efficiency, accelerating glycolysis to compensate for energy demand, and thereby increasing the pyruvate pool. This aligns with the strategy of uncoupling energy metabolism to enhance production [51]. Moreover, this may also reduce intracellular ROS generation and enhance cell tolerance to oxidative stress [52].

Our further characterization revealed that the combined mutations *cobT^V192V^* and *ctaE^L79P^* synergistically enhanced 2,3-BD tolerance. Under 60 g/L stress, K4-*cobT-ctaE* exhibited faster logarithmic growth, higher OD_600_, improved membrane integrity, and greater metabolic viability than CGK4 (Figure 5E–G). Neither single mutation alone had any observable effect, indicating a strong synergistic interaction between the two. This observation underscores a critical physiological trade-off: mutations like *atpG^L250P^* and Δ*ctaD* may drastically reinforce stress defense, possibly by reducing energy coupling efficiency or diminishing proton motive force, which could paradoxically mitigate membrane damage. However, this energetic uncoupling, while beneficial for viability, may come at the cost of reduced ATP yield per glucose consumed, thereby limiting the carbon flux available for the NADH-intensive 2,3-BD synthesis pathway. In contrast, the *cobT^V192V^*-*ctaE^L79P^* combination may modulate cofactor metabolism and fine-tune electron transfer without complete respiratory blockade, presumably preserving reductive power and pyruvate availability while maintaining sufficient resilience. We therefore conclude that industrial strain improvement should prioritize an optimal balance between tolerance and biosynthetic capacity rather than pursue maximal tolerance alone.

## 4. Materials and Methods

### 4.1. Strains and Media

Strains and plasmids used in this study are listed in Supplementary Table S1. *E. coli* DH5α was used for plasmid construction and was grown in LB medium. BHI and CGIII medium [53] were used for the pre-culture and seed culture of *C. glutamicum*, respectively. Solid plate medium consisted of BHI supplemented with 2% (*w*/*v*) agar. CGXIIP medium [18] was used for 2,3-BD tolerance testing in shake flasks and tolerance screening in well plates. Fed-batch fermentation of 2,3-BD was conducted in LBRC-205 medium, which was derived from LBRC medium [53] by reducing corn steep liquor powder from 5% to 2%, and yeast extract from 1% to 0.5% (all *w*/*v*). Glucose was added where appropriate as an initial or supplemental carbon source. Antibiotics were added as described in [54]. Considering the varying cytotoxicity of 2,3-BD stereoisomers [19], only (2*R*,3*R*)-2,3-BD was used here. All subsequent mentions of 2,3-BD tolerance or screening refer to this isomer.

### 4.2. Construction of Plasmids and Strains

Primers used in this study are listed in Supplementary Table S2. All chromosomal recombination in *C. glutamicum* including base substitution and gene deletion was constructed using the suicide plasmid pD-sacB [55].

To introduce mutations into the endogenous gene *atpG*, the plasmid pD-sacB-*atpG* was constructed as follows: the flanking regions of the mutation in the *atpG* locus with exchange of the 749th nucleotide sequence T to C were amplified from genomic DNA of *C. glutamicum* L1 using the primer pairs atpG-F and atpG-R by PCR. Plasmid pD-sacB was linearised by PCR using primers pD-F and pD-R. The DNA fragment containing the allelic replacement site was ligated to the linearized segment of the plasmid using seamless assembly to construct pD-sacB-*atpG*. Other plasmids for nucleotide mutations were constructed analogously.

### 4.3. ARTP Mutagenesis and Screening

Following pre-culture to an OD_600_ of 8–10, a 10-μL sample of a tenfold PBS-diluted cell suspension (10^7^–10^8^ cells/mL) of *C. glutamicum* CGK4 was placed on the mutagenesis stage. A radio frequency power of 120 W was used for the ARTP mutagenesis system (Tmaxtree, Wuxi, China). The working gas was helium (purity ≥ 99.9%), with a flow rate of 10 standard liters per minute (SLM). For lethality rates, the mutagenesis time was selected to be 0, 30, 60, 90, 120, and 150 s. The processed samples were transferred into 990 μL PBS, diluted 10^3^-fold, and then plated onto BHI solid medium and incubated at 30 °C for 48 h to calculate lethality rates.

For the mutant library, the mutagenesis time was 120s. After ARTP mutagenesis, the treated samples were spread on the plates containing BHI solid medium with 8% (*w*/*v*) 2,3-BD. After 7 days of cultivation, large single colonies were randomly selected and inoculated into 24-deep-well plates containing 5 mL CGXIIP medium with 2% (*w*/*v*) glucose and 6% (*w*/*v*) 2,3-BD. After 48 h of cultivation at 500 rpm, the OD_600_ and 2,3-BD concentration were measured. Then the mutants with titer in the top 25% were re-screened using the same procedure, except that each strain was tested in triplicate. All cultures were performed at 30 °C.

### 4.4. Genomic DNA Sequencing

The genomes of the parental strain CGK4 and the mutants L1, L5, and L9 were re-sequenced by GENEWIZ (Suzhou, China) with the Illumina HiseqXten/Novaseq/MGI2000 system, respectively. SNV/InDel calling was performed using Sentieon BWA with the *C. glutamicum* ATCC 13032 reference genome (RefSeq: GCF_000011325.1) from the NCBI database. Other genetic sequencing was performed by Azenta (Tianjin, China).

### 4.5. Culture Conditions

The resuscitation, pre-culture, and seed culture of *C. glutamicum* strains were performed as described in [54].

For 2,3-BD tolerance testing on plates, pre-cultured cells were diluted 10^6^-fold, and 100 μL was spread onto plates containing a gradient of 2,3-BD concentrations (0, 20, 40, 60, 80, and 100 g/L) and incubated for 1 to 10 days. For 2,3-BD tolerance testing in shake flasks, the seed culture was inoculated at an initial OD_600_ of 1 into a 100 mL shake flask containing 25 mL CGXIIP medium with 4% (*w*/*v*) glucose and specific concentrations of 2,3-BD, and grown at 180 rpm for 24 h. The OD_600_ value measured at 24 h was uniformly adopted as the indicator for 2,3-BD tolerance of strains.

For fed-batch fermentation, the seed culture was inoculated at an initial OD_600_ of 1 into a 250 mL shake flask containing 50 mL LBRC-205 medium with 12% (*w*/*v*) glucose and grown at 180 rpm for 120 h. A 100% glucose stock solution was added to maintain its concentration between 1% and 5%.

For verification of genetic stability, mutant strains were cultivated in CGIII medium with 2% (*w*/*v*) glucose and grown at 220 rpm, and 1 mL of the culture was transferred to fresh medium every 12 h. This process was repeated ten times, with the strain from each generation preserved and subsequently subjected to fed-batch fermentation. All strains in shake-flask experiments were tested in triplicate. All cultivations were carried out at 30 °C.

### 4.6. Apoptosis and Viability Assay

One milliliter of pre-cultured seed culture was transferred to a 100-mL shake flask containing 25 mL CGXIIP medium with 0.5% (*w*/*v*) glucose and 60 g/L 2,3-BD, and cultured at 180 rpm and 30 °C. Cells in logarithmic phase (OD_600_ of 1.6–1.8) were harvested by centrifugation at 12,000 rpm and 4 °C for 10 min, washed with PBS, and resuspended. Under dark conditions, 500 μL of the diluted sample was mixed with 500 μL of 20 μg/mL PI or 10 μM FDA, and incubated at 37 °C for 30 min. After washing, fluorescence and absorbance were measured using an Infinite E Plex microplate reader (Tecan, Grödig, Austria). Additional details were obtained from the PI (Yuanye, Shanghai, China) and FDA (Yeasen, Shanghai, China) kit instructions.

### 4.7. Analytical Methods

The bacterial growth was determined by measuring the optical density at 600 nm (OD_600_) using a TU-1901 UV-Vis spectrophotometer (Purkinje General, Beijing, China). The fermentation broth samples were centrifuged at 12,000× *g* for 10 min to collect supernatant. Glucose concentration was measured using an SBA Bio-analyzer (Shandong Academy of Sciences, Jinan, China). Metabolite concentrations were determined by an LC-20AR XR HPLC system (Shimadzu, Kyoto, Japan) using an HPX-87H organic acid analysis column (Bio-Rad, Shanghai, China). The chromatographic separation was performed under the following conditions: isocratic elution with 5 mM H_2_SO_4_ as the mobile phase at a flow rate of 0.5 mL/min, column temperature set to 60 °C, and an injection volume of 10 μL [18,54].

### 4.8. Statistical Analysis

All experiments were independently performed in triplicate. Data are presented as mean ± standard deviation (SD). Standard deviation was calculated using the STDEV function for sample data. Statistical significance was determined by one-way ANOVA followed by Tukey’s post hoc test. Significance levels are denoted as * (*p* < 0.05), ** (*p* < 0.01), and *** (*p* < 0.001). For the compact letter display, means that do not share a letter are significantly different (*p* < 0.05).

## 5. Conclusions

In this study, three *C. glutamicum* mutants with improved 2,3-BD tolerance and production were obtained by ARTP mutagenesis. Multiple beneficial mutations were identified and analyzed through genomics and reverse engineering. By optimizing mutation combinations, the engineered strain K4-*cobT-ctaE* was constructed, which showed significantly enhanced 2,3-BD production. In shake-flask fed-batch fermentation, it produced 84.35 g/L (2*R*,3*R*)-2,3-BD, which was 38.3% higher than that of the parental strain. This study also underscores that the correlation between tolerance and production is nuanced and context-dependent. The most productive strains are not necessarily the most tolerant ones; rather, they exhibit an optimized balance between stress survival and metabolic efficiency. Our findings provide a reference for applying ARTP mutagenesis to improve product tolerance in microbial cell factories and offer new perspectives for 2,3-BD biosynthesis.

## Figures and Tables

**Table 1:** Information on mutations of ARTP mutant strains by whole-genome sequencing.

Strain	Type	Gene	Locus	NT Site	Ref	Obs	AA Change	ExonicFunc	Protein Product
L1	SNV	*CGL_RS06035*	*atpG*	749	T	C	L250P	NS	F_0_F_1_ ATP synthase subunit gamma
L1	SNV	*CGL_RS07320*	*thiD1*	1597	A	G	T533A	NS	bifunctional hydroxymethylpyrimidine kinase/phosphomethylpyrimidine kinase
L1	SNV	*CGL_RS09185*	*Cgl1844*	1943	T	C	L648S	NS	DEAD/DEAH box helicase family protein
L1	SNV	*CGL_RS10920*	*cobT*	576	T	C	V192V	SS	nicotinate-nucleotide--dimethylbenzimidazole phosphoribosyltransferase
L5	InDel	*CGL_RS08870*	*cglIR*	959	A	-	E320fs	FD	restriction endonuclease PLD domain-containing protein
L5	SNV	*CGL_RS02360*	*Cgl0464*	41	G	A	R14Q	NS	Type 1 glutamine amidotransferase-like domain-containing protein
L5	SNV	*CGL_RS03380*	*trpS*	265	A	G	T89A	NS	tryptophan--tRNA ligase
L5	SNV	*CGL_RS09580*	*oxyR*	633	T	C	I1211I	SS	hydrogen peroxide-inducible genes activator
L5	SNV	*CGL_RS09930*	*Cgl1996*	488	A	T	T150S	NS	VWA domain-containing protein
L5	SNV	*CGL_RS10675*	*Cgl2150*	546	C	A	H128Q	NS	DivIVA domain-containing protein
L5	SNV	*CGL_RS10785*	*idsA*	622	A	G	K208E	NS	geranylgeranyl pyrophosphate synthase IdsA
L5	SNV	*CGL_RS10885*	*ctaE*	236	T	C	L79P	NS	heme-copper oxidase subunit III
L5	SNV	*CGL_RS10895*	*ctaC*	660	T	C	D220D	SS	cytochrome c oxidase subunit II
L5	SNV	*CGL_RS12190*	*Cgl2460*	727	T	A	F243I	NS	sugar ABC transporter permease
L5	SNV	*CGL_RS15110*	*trpCF*	1390	A	G	K464E	NS	bifunctional indole-3-glycerol-phosphate synthase TrpC/phosphoribosylanthranilate isomerase TrpF
L9	SNV	*CGL_RS03665*	*Cgl0735*	2253	T	C	L506L	SS	ATP-dependent helicase
L9	SNV	*CGL_RS09515*	*Cgl1912*	456	G	A	M152I	NS	DUF418 domain-containing protein
L9	SNV	*CGL_RS11210*	*Cgl2264*	40	A	C	T14P	NS	hypothetical protein
L9	InDel	*CGL_RS12540*	*ctaD*	570–571	GC	-	M190fs	FD	cytochrome c oxidase subunit I

NS: nonsynonymous SNV; SS: synonymous SNV; FD: frameshift deletion.

**Table 2:** Summary of literature on microbial fermentation for the optically pure (2*R*,3*R*)-2,3-butanediol production.

Hosts	Substrates	Fermentation Mode and Scale	Titer (g/L)	Purity (%)	Yield (g/g)	Productivity (g/L/h)	Ref.
*Klebsiella pneumoniae*	Crude glycerol	Fed-batch, 5 L fermentor	89.47	98	0.35	1.24	[8]
*Klebsiella oxytoca*	Glucose	Fed-batch, 2 L fermentor	106.7	92	0.40	3.1	[33]
*Enterobacter cloacae*	Glucose and xylose	Fed-batch, 5 L fermentor	152.0	97.5	0.49	3.5	[34]
	Corn stover hydrolysate		119.4	96	0.48	2.3	
*Bacillus licheniformis*	Glucose	Batch, 500 mL shake flask	38.91	99.4	0.508	NM	[35]
		Fed-batch, 5 L fermentor	123.7	99	NM	2.95	
*Bacillus velezensis*	Soluble inulin	Batch, 1 L fermentor	96.4	86.2	NM	0.8	[36]
*Bacillus subtilis*	Glucose	Fed-batch, shake flask	58.8	NM	0.35	NM	[37]
		Fed-batch, 5 L fermentor	96.5	94.7	NM	1.34	
*Paenibacillus polymyxa*	Glucose	Fed-batch, 5 L fermentor	77.3	99	NM	NM	[38]
*Paenibacillus polymyxa*	Sucrose	Fed-batch, 2 L fermentor	111	98	0.48	2.06	[39]
*S. cerevisiae*	Glucose and galactose	Fed-batch, 5 L fermentor	100	98	0.35	0.33	[40]
*S. cerevisiae*	Glucose	Fed-batch, 1 L fermentor	178	NM	NM	2.64	[13]
	Cassava hydrolysate		132	NM	NM	1.92	
*C. glutamicum*	Glucose	Fed-batch, 5 L fermentor	144.9	98	0.429	1.10	[18]
*C. glutamicum*	Glucose	Fed-batch, 250 mL shake flask	84.35	98	0.447	0.7	This study
*E. coli*	Glucose	Batch, 250 mL shake flask	33.8	99	0.42	2.11	[15]
		Fed-batch, 3 L fermentor	115	99	0.42	1.44	
*E. coli*	Glucose	Fed-batch, 3 L fermentor	88.0	NM	0.35	1.87	[16]

NM: not mentioned.

## Data Availability

The original contributions presented in this study are included in the article/Supplementary Materials. Further inquiries can be directed to the corresponding authors.

## Supplementary Materials

The following supporting information can be downloaded at https://www.mdpi.com/article/10.3390/molecules31152714/s1, Figure S1: Growth of CGK4 on plates exposed to increasing 2,3-butanediol stress. The 2,3-butanediol concentrations and corresponding observation days were as follows: (a) 0 g/L, 2 d; (b) 20 g/L, 2 d; (c) 40 g/L, 3 d; (d) 60 g/L, 5 d; (e) 80 g/L, 7 d; and (f) 100 g/L, 10 d; Figure S2: Effect of radiation time on the lethality of strain CGK4 in ARTP mutagenesis. The working gas was helium at a flow rate of 10 SLM, and the irradiation power was 120 W. Values and error bars represent mean and SD (*n* = 3), respectively; Figure S3: ARTP mutant screening and validation. (A) Primary screening using 24-deep well plates. (B) Secondary screening using 24-deep well plates. Values and error bars represent mean and SD (*n* = 3), respectively; Figure S4: Fermentation evaluation of elite mutants. Shake-flask fed-batch fe4ntation profiles of strains (A) L1, (B) L5, and (C) L9. (D) Production stability of L1, L5, and L9. Values and error bars represent mean and SD (*n* = 3), respectively; Table S1: Strains and plasmids used in this study; Table S2: Primers used in this study.

## Abbreviations

The following abbreviations are used in this manuscript:

ARTP	atmospheric and room temperature plasma
2,3-BD	2,3-butanediol
GRAS	generally recognized as safe
*C. glutamicum*	*Corynebacterium glutamicum*
ALE	adaptive laboratory evolution
*S. cerevisiae*	*Saccharomyces cerevisiae*
*E. coli*	*Escherichia coli*
PI	propidium iodide
FDA	fluorescein diacetate
RFU	relative fluorescence units
ROS	reactive oxygen species
ATP	adenosine triphosphate
