# Supplementary material for: Mutagenesis and Analysis for Enhancing (2R,3R)-2,3-Butanediol Tolerance and Production in Corynebacterium glutamicum
Source: Molecules. 2026 Aug 4;31(15):2714. doi: 10.3390/molecules31152714 (PMC13467605; doi:10.3390/molecules31152714)
Supplement: Supplementary file 1 [file molecules-31-02714-s001.zip › molecules-4435220-supplementary.pdf]

Supplementary Materials

Mutagenesis and Analysis for Enhancing (2*R*,3*R*)-2,3-Butanediol Tolerance and Production in *Corynebacterium glutamicum*

Zilong Liu [1,2,3,†], Haoran Cui [1,2,3,†], Yuxuan Jiang [1,2,3], Zhiwen Wang [1,3,4,*] and Tao Chen [1,2,3,*]

[1] State Key Laboratory of Synthetic Biology, Tianjin University, Tianjin 300072, China; lzl16630736892@163.com (Z.L.); cuihaoran@tju.edu.cn (H.C.); yuxuan_jiang@tju.edu.cn (Y.J.)
[2] Frontier Science Center for Synthetic Biology and Key Laboratory of Systems Bioengineering (Ministry of Education), Tianjin University, Tianjin 300072, China
[3] School of Synthetic Biology and Biomanufacturing, Tianjin University, Tianjin 300072, China
[4] Key Laboratory of Ministry of Education for Protection and Utilization of Special Biological Resources in Western China, School of Life Sciences, Ningxia University, Yinchuan 750021, China
* Correspondence: zww@nxu.edu.cn (Z.W.); chentao@tju.edu.cn (T.C.)
† These authors contributed equally to this work.

List of content

Supplementary Figure S1. Growth of CGK4 on plates exposed to increasing 2,3-butanediol stress

Supplementary Figure S2. Effect of radiation time on the lethality of strain CGK4 in ARTP mutagenesis

Supplementary Figure S3. ARTP mutant screening and validation

Supplementary Figure S4. Fermentation evaluation of elite mutants

Supplementary Table S1. Strains and plasmids used in this study

Supplementary Table S2. Primers used in this study

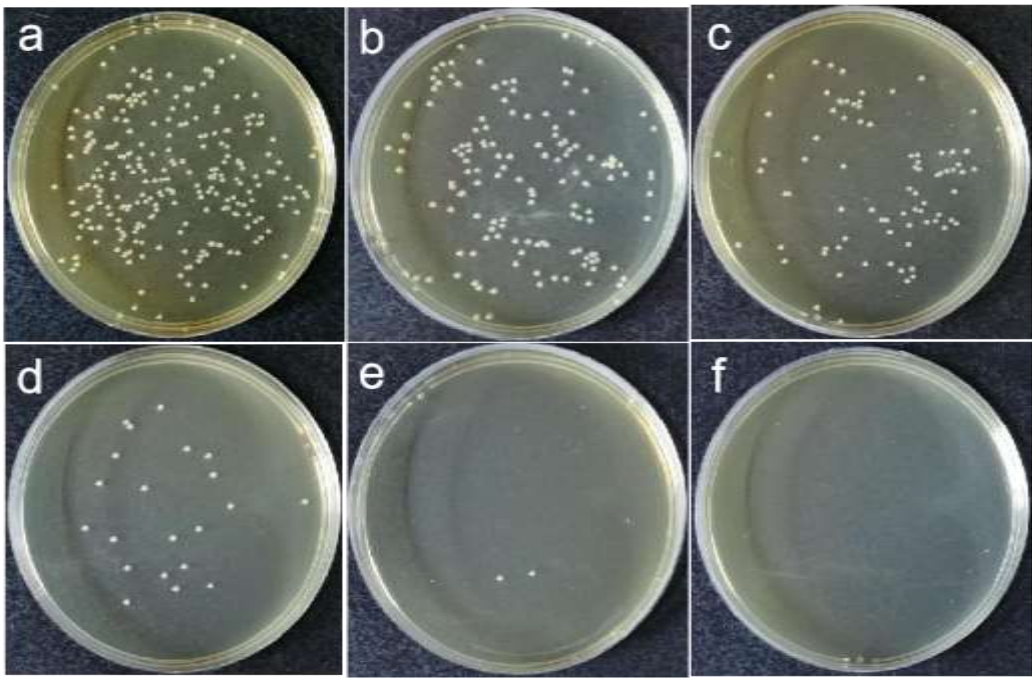

Figure S1. Growth of CGK4 on plates exposed to increasing 2,3-butanediol stress.

The 2,3-butanediol concentrations and corresponding observation days were as follows: (a) 0 g/L, 2 d; (b) 20 g/L, 2 d; (c) 40 g/L, 3 d; (d) 60 g/L, 5 d; (e) 80 g/L, 7 d; and (f) 100 g/L, 10 d.

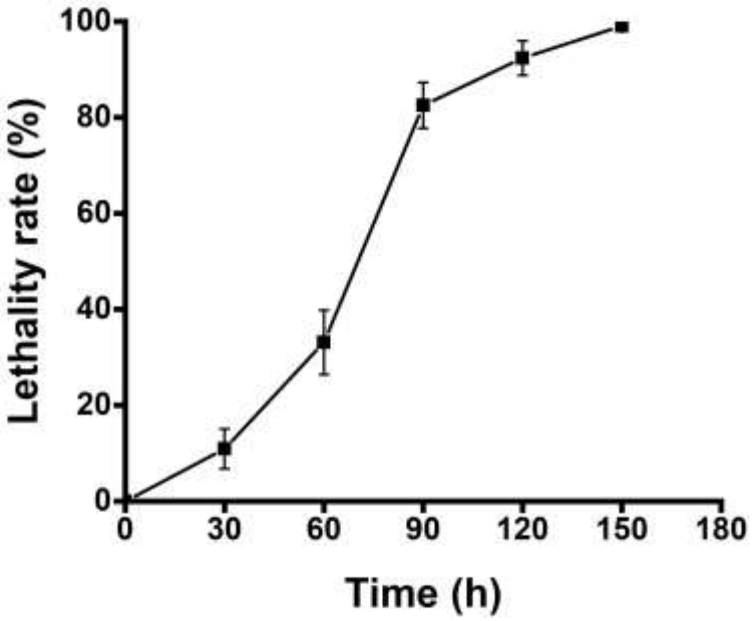

Figure S2. Effect of radiation time on the lethality of strain CGK4 in ARTP mutagenesis. The working gas was helium at a flow rate of 10 SLM, and the irradiation power was 120 W. Values and error bars represent mean and SD (n = 3), respectively.

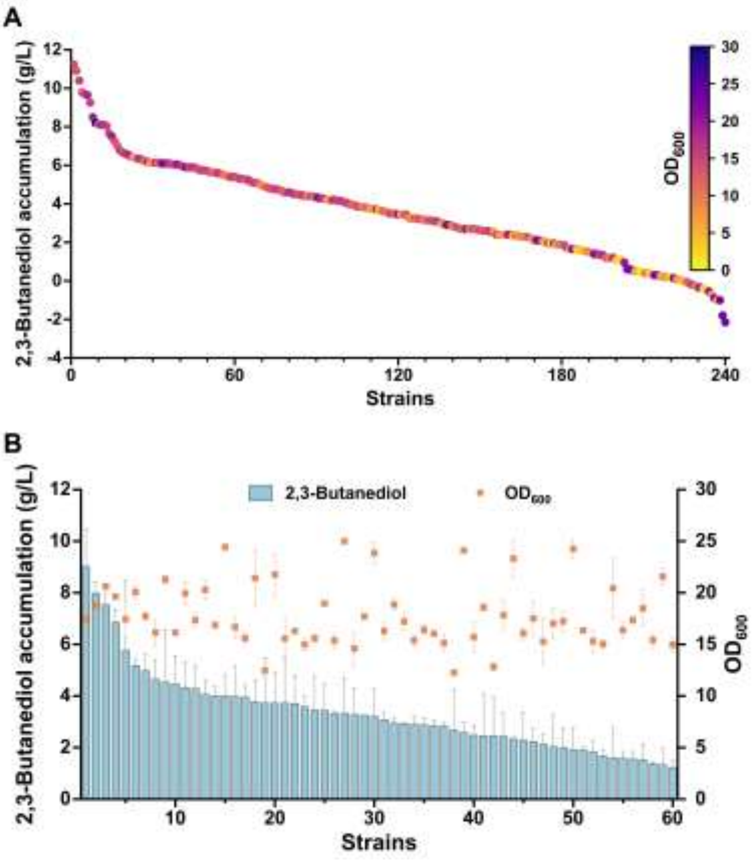

Figure S3. ARTP mutant screening and validation. (A) Primary screening using 24-deep well plates. (B) Secondary screening using 24-deep well plates. Values and error bars represent mean and SD (n = 3), respectively.

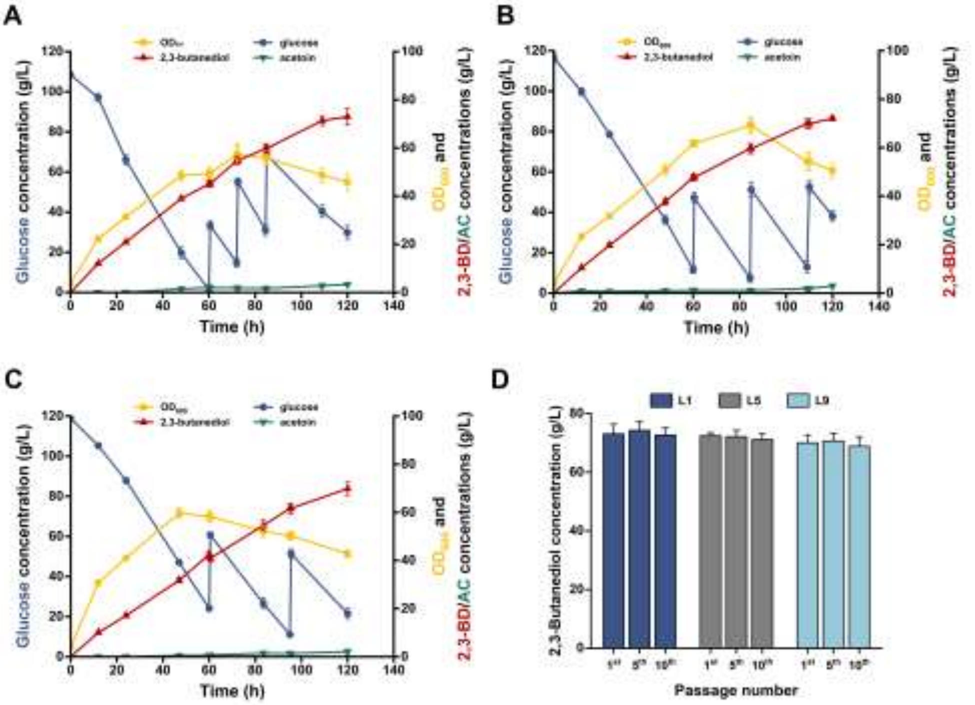

Figure S4. Fermentation evaluation of elite mutants. Shake-flask fed-batch fe4ntation profiles of strains (A) L1, (B) L5, and (C) L9. (D) Production stability of L1, L5, and L9. Values and error bars represent mean and SD (*n* = 3), respectively.

Table S1. Strains and plasmids used in this study.

Strain/plasmid	Relevant characteristics	References
E. coli DH5α	Host for plasmid construction	Lab stock
C. glutamicum ATCC13032	*C. glutamicum* wild type<br>Biotin auxotrophic	Lab stock
CGK4	ATCC13032Δ*pta*Δ*ack*Δ*butA*Δ*nagD*Δ*ppc*,Δ*ackA*::P_{tuf}-*alsSD*,Δ*butA*::P_{tuf}-*alsSD*,Δ*nagD*::P_{tuf}-*alsSD*,P_1-*gltA*,Δ*ldh*::P_{sod}-*bdhA*-*udhA*,$atpG^{T817C,C818T}$	Lab stock
L1	CGK4 derived strain, mutated strain,containing four mutation sites	This study
L5	CGK4 derived strain, mutated strain,containing eleven mutation sites	This study
L9	CGK4 derived strain, mutated strain，containing four mutation sites	This study
K4-*atpG*	CGK4 $atpG^{T749C}$	This study
K4-*thiD1*	CGK4 $thiD1^{A1597G}$	This study
K4-*Cgl1844*	CGK4 $Cgl1844^{T1934C}$	This study
K4-*cobT*	CGK4 $cobT^{T576C}$	This study
K4-*cglIR*	CGK4 $cglIR^{A959del}$	This study
K4-*Cgl0464*	CGK4 $Cgl0464^{G41A}$	This study
K4-*trpS*	CGK4 $trpS^{A265G}$	This study
K4-*oxyR*	CGK4 $oxyR^{T633C}$	This study
K4-*Cgl1996*	CGK4 $Cgl1996^{A488T}$	This study
K4- *Cgl2150*	CGK4 $Cgl2150^{C546A}$	This study
K4-*idsA*	CGK4 *idsA*	This study
K4-*ctaE*	CGK4 $ctaE^{T236C}$	This study
K4-*ctaC*	CGK4 $ctaC^{T660C}$	This study
K4-*Cgl2460*	CGK4 $Cgl2460^{T727A}$	This study
K4-*trpCF*	CGK4 $trpCF^{A1390G}$	This study
K4-*Cgl0735*	CGK4 $Cgl0735^{T2253C}$	This study
K4-*Cgl1912*	CGK4 $Cgl1912^{G525A}$	This study
K4-*Cgl2264*	CGK4 $Cgl2264^{A40C}$	This study
K4-Δ*ctaD*	CGK4 Δ*ctaD*	This study
L1-*Cgl0464*	L1 $Cgl0464^{G41A}$	This study
L1-*ctaE*	L1 $ctaE^{T236C}$	This study

Strain/plasmid	Relevant characteristics	References
L1-*Cgl0735*	L1 *Cgl0735*^{T2253C}	This study
L1-*Cgl1912*	L1 *Cgl1912*^{G525A}	This study
L1-*ctaD*	L1 Δ*ctaD*	This study
K4-*atpG-cobT*	K4 *atpG*^{T749C}, *cobT*^{T576C}	This study
K4-*cobT-ctaE*	K4 *cobT*^{T576C}, *ctaE*^{T236C}	This study
K4-*atpG-ctaE*	K4 *atpG*^{T749C}, *ctaE*^{T236C}	This study
pD-*sacB*	Kan^{R}; vector for in-frame deletion (*sacB*$_{B.sub.}$; *lacZα*; *OriV*$_{E.c.}$)	Lab stock
pD-*sacB-atpG*^{T749C}	Kan^{R}, containing the sequence for *atpG* exchange T749C	This study
pD-*sacB-thiD1*^{A1597G}	Kan^{R}, containing the sequence for *thiD1* exchange A1597G	This study
pD-*sacB-Cgl1844*^{T1934C}	Kan^{R}, containing the sequence for *Cgl1844* exchange T1934C	This study
pD-*sacB-cobT*^{T576C}	Kan^{R}, containing the sequence for *cobT* exchange T576C	This study
pD-*sacB-cglIR*^{A959del}	Kan^{R}, containing the sequence for *cglIR* deletion A959	This study
pD-*sacB-Cgl0464*^{G41A}	Kan^{R}, containing the sequence for *Cgl0464* exchange G41A	This study
pD-*sacB-trpS*^{A265G}	Kan^{R}, containing the sequence for *trpS* exchange A265G	This study
pD-*sacB-oxyR*^{T633C}	Kan^{R}, containing the sequence for *oxyR* exchange T633C	This study
pD-*sacB-Cgl1996*^{A448T}	Kan^{R}, containing the sequence for *Cgl1996* exchange A448T	This study
pD-*sacB*- *Cgl2150*^{C546A}	Kan^{R}, containing the sequence for *Cgl2150* exchange C546A	This study
pD-*sacB-idsA*^{A622G}	Kan^{R}, containing the sequence for *idsA* exchange A622G	This study
pD-*sacB-ctaE*^{T236C}	Kan^{R}, containing the sequence for *ctaE* exchange	This study

**Strain/plasmid**	**Relevant characteristics**	**References**
	T236C	
pD-*sacB*-*ctaC*^{T660C}	Kan^R, containing the sequence for *ctaC* exchange T660C	This study
pD-*sacB*-*Cgl2460*^{T727A}	Kan^R, containing the sequence for *Cgl2460* exchange T727A	This study
pD-*sacB*-*trpCF*^{A1390G}	Kan^R, containing the sequence for *trpCF* exchange A1390G	This study
pD-*sacB*-*Cgl0735*^{T2253C}	Kan^R, containing the sequence for *Cgl0735* exchange T2253C	This study
pD-*sacB*-*Cgl1912*^{G525A}	Kan^R, containing the sequence for *Cgl1912* exchange G525A	This study
pD-*sacB*-*Cgl2264*^{A40C}	Kan^R, containing the sequence for *Cgl2264* exchange A40C	This study
pD-sacB-Δ*ctaD*	Kan^R, containing the sequence for *ctaD* knockout	This study

Table S2. Primers used in this study.

Primer	Sequence	Purpose
pD-F	AAGCTTGGCACTGGCCGTCGTTTTACAACGTCGTGACTG	To linearly amplify pD-*sacB*
pD-R	GAATTCGTAATCATGTCATAGCTGTTTCCTGT	To linearly amplify pD-*sacB*
atpG-F	GACATGATTACGAATTCGCGCTTTCCAGACCACCGATG	To construct pD-*sacB*-$atpG^{T749C}$
atpG-R	CGACGGCCAGTGCCAAGCTTCCTCAGAACCTGCCTGGGT	To construct pD-*sacB*-$atpG^{T749C}$
thiD1-F	TATGACATGATTACGAATTCCACAATGCATACGCAAGGACAGG	To construct pD-*sacB*-$thiD1^{A1597G}$
thiD1-R	CGACGGCCAGTGCCAAGCTTGAACTCGCTGCTACGCCCATCGAC	To construct pD-*sacB*-$thiD1^{A1597G}$
Cgl1844-F	TATGACATGATTACGAATTCGATACTTTGCATGTTTTACGTATAC	To construct pD-*sacB*-$Cgl1844^{T1934C}$
Cgl1844-R	CGACGGCCAGTGCCAAGCTTTAGAGCGCGATACCTGCGTGGTC	To construct pD-*sacB*-$Cgl1844^{T1934C}$
cobT-F	TATGACATGATTACGAATTCCTGGGCACCTGGCTCACGCACGCCAC	To construct pD-*sacB*-$cobT^{T576C}$
cobT-R	CGACGGCCAGTGCCAAGCTTAGAAGGCGTTGTGGTTTTTCAGT	To construct pD-*sacB*-$cobT^{T576C}$
cglIR-F	TATGACATGATTACGAATTCGTTAATGTTGTGTTCAATGC	To construct pD-*sacB*-$cglIR^{A959del}$
cglIR-R	CGACGGCCAGTGCCAAGCTTGAAGTTTGAACAGTGCTGC	To construct pD-*sacB*-$cglIR^{A959del}$
Cgl0464-F	ATGACATGATTACGAATTCGTCGAAGGATCCCACGTCAG	To construct pD-*sacB*-$Cgl0464^{G41A}$
Cgl0464-R	CGACGGCCAGTGCCAAGCTTGCAACTCGCGCGATGTCATCAG	To construct pD-*sacB*-$Cgl0464^{G41A}$
trpS-F	ATGACATGATTACGAATTCTCGCAGTGGCGCTGGAAAT	To construct pD-*sacB*-$trpS^{A265G}$
trpS-R	GACGGCCAGTGCCAAGCTTCGTCGGTATAACGGTCTTGT	To construct pD-*sacB*-$trpS^{A265G}$
oxyR-F	TATGACATGATTACGAATTCCGTGGACCAGAGTTCTTC	To construct pD-*sacB*-$oxyR^{T633C}$
oxyR-R	CGACGGCCAGTGCCAAGCTTCCATCAATGTTGGTCAAAC	To construct pD-*sacB*-$oxyR^{T633C}$
Cgl1996-F	TATGACATGATTACGAATTCTACTCGCTGACCTGCAAGCTTTGG	To construct pD-*sacB*-$Cgl1996^{A448T}$
Cgl1996-R	CGACGGCCAGTGCCAAGCTTCTGAGCAGTTCATGGCTAAGTGGG	To construct pD-*sacB*-$Cgl1996^{A448T}$
Cgl2150-F	TATGACATGATTACGAATTCGATTGCCGGATCATAACCTC	To construct pD-*sacB*-$Cgl2150^{C546A}$
Cgl2150-R	CGACGGCCAGTGCCAAGCTTGGTAACGAATTGATATAGGTG	To construct pD-*sacB*-$Cgl2150^{C546A}$
idsA-F	TATGACATGATTACGAATTCGGGTTGATCCGCCAGCACCATAAGT	To construct pD-*sacB*-$idsA^{A622G}$
idsA-R	GACGGCCAGTGCCAAGCTTGTGTTGCGCCAATCATGAGAGACCTC	To construct pD-*sacB*-$idsA^{A622G}$
ctaE-F	TATGACATGATTACGAATTCGTCCTGGGCCACCGCCATTAGCT	To construct pD-*sacB*-$ctaE^{T236C}$
ctaE-R	CGACGGCCAGTGCCAAGCTTCTTCAGCCCAAGCTCCATTT	To construct pD-*sacB*-$ctaE^{T236C}$
ctaC-F	TATGACATGATTACGAATTCCTGCCCTGTTGGATAGTTTC	To construct pD-*sacB*-$ctaC^{T660C}$

Primer	Sequence	Purpose
ctaC-R	CGACGGCCAGTGCCAAGCTTGTGGCATACACCAATTGCCTAG	To construct pD-*sacB*-*ctaC*^{T660C}
Cgl2460-F	TATGACATGATTACGAATTCATTGGATGACCACCGGGATGGTCTT	To construct pD-*sacB*-*Cgl2460*^{T727A}
Cgl2460-R	CGACGGCCAGTGCCAAGCTTGAGGACGTCGTCAAGCACTTCAAT	To construct pD-*sacB*-*Cgl2460*^{T727A}
trpCF-F	TATGACATGATTACGAATTCGCTTGATGATGAAGAGTAC	To construct pD-*sacB*-*trpCF*^{A1390G}
trpCF-R	CGACGGCCAGTGCCAAGCTTCTCTACACCTTCATCGTCAATG	To construct pD-*sacB*-*trpCF*^{A1390G}
Cgl0735-F	TATGACATGATTACGAATTCGAGCTTGGAGTTACGGATGAATTTC	To construct pD-*sacB*-*Cgl0735*^{T2253C}
Cgl0735-R	GACGGCCAGTGCCAAGCTTGATCATTTCCGCAGATCCCGGC	To construct pD-*sacB*-*Cgl0735*^{T2253C}
Cgl1912-F	TATGACATGATTACGAATTCTCATCTGGAGGCGCCGATGTGGAAAG	To construct pD-*sacB*-*Cgl1912*^{G525A}
Cgl1912-R	CGACGGCCAGTGCCAAGCTTCTTACGGATCGGGCTATACCGGAG	To construct pD-*sacB*-*Cgl1912*^{G525A}
Cgl2264-F	TATGACATGATTACGAATTCTGGTGGGAATTGCTTACGCCCGTTT	To construct pD-*sacB*-*Cgl2264*^{A40C}
Cgl2264-R	GACGGCCAGTGCCAAGCTTTGTGGCGTCGGGAATTGGGGTCAG	To construct pD-*sacB*-*Cgl2264*^{A40C}
ctaD-U-F	CTATGACATGATTACGAATTCTCCTCGAAATCGAGCTCACCG	To construct pD-sacB-Δ*ctaD*
ctaD-U-R	GTCCCGTAGTCAGGAGGAACCTAAGCGTCTGATTTAAGTCGGTACCTGAC	To construct pD-sacB-Δ*ctaD*
ctaD-D-F	TACCGACTTAAATCAGACGCTTAGGTTCCTCCTGA*CTAC*GGGACG	To construct pD-sacB-Δ*ctaD*
ctaD-D-R	GACGGCCAGTGCCAAGCTTGCTGACCAACACCGCCGACGTGAT	To construct pD-sacB-Δ*ctaD*
